# Bullet Free in Pleural Cavity

**Published:** 1916-10

**Authors:** 


**Bullet Free in Pleural Cavity.** Pl. Mauclaire, Soc. Chir.,

Nov. 3, 1915, reports the case of a soldier, struck in the right buttock, the ball passing obliquely in front of the spinal column into the left pleural cavity. An artificial pneumothorax was created after the method of Forlanini and, 3 weeks later, the ball was removed by the finger, and forceps, from the cul de sac.

